# Autophagy: a critical mechanism of N^6^-methyladenosine modification involved in tumor progression and therapy resistance

**DOI:** 10.1038/s41419-024-07148-w

**Published:** 2024-10-28

**Authors:** Feiyang Wang, Qiudi Liao, Zihao Qin, Jingyi Li, Qingqing Wei, Mengna Li, Hongyu Deng, Wei Xiong, Ming Tan, Ming Zhou

**Affiliations:** 1 NHC Key Laboratory of Carcinogenesis, Hunan Key Laboratory of Oncotarget Gene, the Affiliated Cancer Hospital of Xiangya School of Medicine, Central South University/ Hunan Cancer Hospital, Changsha, China; 2 Xiangya School of Medicine, Central South University, Changsha, China; 3 Cancer Research Institute and School of Basic Medical Sciences, Central South University, Changsha, China; 4 The Key Laboratory of Carcinogenesis and Cancer Invasion of the Chinese Ministry of Education, Central South University, Changsha, China; 5 Graduate Institute of Biomedical Sciences and Research Center for Cancer Biology, China Medical University, Taichung, Taiwan

**Keywords:** Cancer genomics, Cancer

## Abstract

N^6^-Methyladenosine (m^6^A) is an evolutionarily highly conserved epigenetic modification that affects eukaryotic RNAs, especially mRNAs, and m^6^A modification is commonly linked to tumor proliferation, progression, and therapeutic resistance by participating in RNA metabolism. Autophagy is an intracellular degradation and recycling biological process by which cells remove damaged organelles, protein aggregates, and other intracellular wastes, and release nutrients to maintain cell survival when energy is scarce. Recent studies have shown that m^6^A modification plays a critical role in the regulation of autophagy, affecting the initiation of autophagy, the formation and assembly of autophagosomes, and lysosomal function by regulating critical regulatory molecules involved in the process of autophagy. Moreover, autophagy can also affect the expression of the three types of regulators related to m^6^A, which in turn affects the levels of their target genes via m^6^A modification. Thus, m^6^A modification and autophagy form a sophisticated regulatory network through mutual regulation, which plays an important role in tumor progression and therapeutic resistance. In this manuscript, we reviewed the effects of m^6^A modification on autophagy as well as the effects of autophagy on m^6^A modification and the roles of the m^6^A-autophagy axis in tumor progression and therapy resistance. Additionally, we summarized the value and application prospects of key molecules in the m^6^A-autophagy axis in tumor diagnosis and therapy.

## Facts

- m^6^A modification affects the initiation of autophagy, the formation and assembly of autophagosomes, and lysosomal function by regulating some key autophagy-related proteins.
- Autophagy can affect the expression of the three types of regulators related to m^6^A, which in turn affects the levels of their target genes via m^6^A modification.
- m^6^A-autophagy axis plays a dual role in tumor progression and therapy resistance.
- m^6^A-autophagy axis-related proteins are potential molecular targets for tumor diagnosis and therapy.

## Open questions

- What roles do m6A modifications and their regulators play in autophagy?
- What mechanism does m6A modification regulate autophagy?
- Can autophagy feedback regulate the expression of m6A regulators and the m6A modification process?
- What role does the m6A-autophagy axis play in the malignant progression and treatment resistance of tumors?
- Can targeting m6A-autophagy axis become a critical molecular strategy for tumor diagnosis and treatment?

## Introduction

Cancer is a major global health problem globally and is expected to rank as the leading cause of death and the most important barrier to increasing life expectancy in every country of the world in the 21st century [1]. Thus, revealing the molecular mechanisms of tumorigenesis and progression is a major scientific challenge that needs to be resolved for tumor diagnosis and prevention. N^6^-Methyladenosine (m^6^A), which refers to the addition of a methyl group to the sixth carbon of adenosine, is the most prevalent internal modification that exists of eukaryotic RNAs, especially on messenger RNA (mRNA) [2, 3]. These m^6^A modifications on mRNAs function as dynamic markers and are regulated by three regulators, “writers”, “erasers” and “readers”, of which “writers” are methyltransferases, “erasers” are demethyltransferases, and “readers” are m^6^A selective binding proteins [4, 5]. The former two can add or remove m^6^A modifications to specific mRNA sequences [6–8], while the latter regulates cellular processes such as cell self-renewal, differentiation, invasion, and apoptosis by selectively recognizing methylated RNA and dynamically regulating its stability, localization or translation [5, 9–11]. Autophagy is an intracellular degradation and recycling biological process that allows cells to remove damaged organelles, protein aggregates, and other intracellular waste while releasing nutrients to sustain cell survival during energy shortages [12]. Autophagy is usually induced by oxygen stress, deprivation of energy or amino acids, irradiation, or drugs [13]. Autophagy can be divided into three main categories, including microautophagy, chaperone-mediated autophagy, and macroautophagy. In addition to nonselective bulk degradation, autophagy specifically recycles organelles such as mitochondria, peroxisomes, ribosomes, endoplasmic reticulum, lysosomes, nuclei, proteasomes, and lipid droplets through selective pathways [14]. In most tumors, autophagy promotes tumorigenesis by regulating mitochondrial quality control and the supply of nutrients needed for cancer cell growth under conditions of nutrient deprivation [15]. However, autophagy may also inhibit tumor growth by inhibiting oxidative stress, inflammation, p62 accumulation, and genome instability [16–18].

Recent studies have shown that m^6^A modifications play an important role in the regulation of autophagy by affecting key regulatory proteins involved in autophagy, thus influencing the initiation of autophagy, the formation and assembly of autophagosomes, and the function of lysosomes. Moreover, autophagy can also affect the expression of the three types of regulatory proteins related to m^6^A, thereby affecting the m^6^A modification levels of target genes. Therefore, m^6^A modifications and autophagy form a precise regulatory network through mutual regulation, playing an important role in tumor progression and the development of therapy resistance. In this manuscript, we reviewed the effects of m^6^A modification on autophagy as well as the effects of autophagy on m^6^A modification and the role of the m^6^A-autophagy axis in in tumor progression and therapy resistance. We also summarizing the value and application prospects of key proteins in the m^6^A-autophagy axis in tumor diagnosis and therapy.

## m^6^A modifications regulate autophagy

### m^6^A writers and autophagy

Enzymes that catalyzes the formation of m^6^A modification are known as writers and were first purified as a protein complex by Bokar et al. in 1994 [19, 20]. Methyltransferase-like 3 (METTL3) was further identified as one of the components of methyltransferase complexes (MTCs) [21]. It uses S-adenosylmethionine (SAM) as the methyl donor to catalyze the transfer of methyl groups to the adenine of the target RNA [22, 23]. An increasing amount of researches indicate that m^6^A is modified through MTCs [24], of which METTL3 serves as the catalytic subunit for the methyltransferase reaction, while methyltransferase-like 14 (METTL14) acts as the RNA-binding scaffold [25]. Similar to METTL3, METTL14 can exhibit weak methyltransferase activity in vitro, but experiments have shown that the two enzymes have higher catalytic activity when combined into a complex [26, 27]. Wilms tumor 1-associated protein (WTAP) can interact with both METTL3 and METTL14 to regulate the level of m^6^A modification [26, 28]. Recent studies have shown that writer-mediated m^6^A modification plays an important role in autophagy regulation by affecting the expression or stability of key proteins associated with autophagy initiation, autophagosome assembly and lysosomal function.

#### METTL3

As the primary catalytic unit, METTL3 can increase autophagic flux through various pathways, and numerous studies have confirmed that increased levels of METTL3 in cells promote autophagy. For example, under nutrient deficiency conditions, METTL3 causes m^6^A methylation of Forkhead box O3 (FOXO3) mRNA in the 3′ untranslated region (3′ UTR) region and increases its stability or translation in a YTH domain family protein 1/3 (YTHDF1/3)-dependent manner, thereby altering the expression of autophagy-related genes downstream of FOXO3, such as ATG3, ATG5, ATG7, ATG10, ATG12, ATG13, ATG14 [29, 30]. METTL3 can also facilitate the m^6^A modification of nucleobindin 1 (NUCB1) mRNA, promote the degradation of NUCB1 mRNA and reduce its translation through YTHDF2, thus reducing activating transcription factor 6 (ATF6) activity and promoting autophagy [31]. Phosphatase and tensin homolog (PTEN) is a tumor suppressor gene, and its mRNA stability is regulated by LINC00470/METTL3-mediated m^6^A modification, resulting in decreased stability of PTEN mRNA and activation of the protein kinase B (AKT) pathway, thereby inhibiting the cellular autophagy process [32]. METTL3 also increases the expression of ZNFX1 antisense RNA1(ZFAS1) by increasing its m^6^A level. It reduces miR-100-3p and promotes ATG10 expression, leading to the inhibition of phosphoinositide 3-kinase(PI3K)/ AKT pathway activation and thereby promoting autophagy [33]. Furthermore, hypoxic conditions lead to the upregulation of aspartyl-tRNA synthetase 1 antisense 1 (DARS-AS1), which recruits METTL3 and METTL14 to mediate m^6^A modifications that increase the translation of DARS, promoting the expression of ATG5 and ATG3 and thus facilitating protective autophagy in cervical cancer cells [34]. Moreover, ubiquitin-specific protease 13(USP13) can stabilize METTL3 by causing its deubiquitination and promote m^6^A modification and stability of ATG5 mRNA depending via insulin growth factor 2 mRNA-binding protein 3 (IGF2BP3), thereby promoting autophagy in osteosarcoma (OS) [35]. METTL3 can also inhibit autophagy by increasing the stability of PSMA3-AS1 mRNA to increase the level of ATG16L1 [36].

#### METTL14

As an enzyme from the same family as METTL3, METTL14 functions in a similar manner to METTL3. In oral squamous cell carcinoma (OSCC), METTL14 mediates the expression of eukaryotic translation initiation factor gamma 1 (eIF4G1) through m^6^A modification and regulates the autophagy levels and biological functions of OSCC cells [37]. Further studies have shown that METTL14 can also promote the expression of the autophagy-related gene RB1 inducible coiled-coil 1(RB1CC1) in an IGF2BP2-dependent manner, thereby increasing autophagy and affecting malignant progression in OSCC [38].

#### WTAP

WTAPs are a regulatory group whose function is to recruit m^6^A MTCs (i.e. METTL3 and METTL14) to target mRNAs [39]. Studies have also shown that WTAP can directly regulate autophagy. It suppresses the expression of the downstream gene Filamin A (FLNA) by regulating its m^6^A modification in the 3’ UTR, thereby further inhibiting autophagy [40]. It can also promote the m^6^A modification of the key phosphorylating enzyme liver kinase B1 (LKB1) mRNA, reducing its stability and expression, which in turn reduces the phosphorylation of AMP-activated protein kinase (AMPK) and inhibits autophagy [41]. In addition, recent studies have demonstrated that WTAP affects the expression of ATG5 in a m^6^A-dependent manner, thus promoting autophagy [42]. WTAP also stabilizes and positively regulates the expression of 3′-UTR region of autophagy-activated kinase 1(ULK1) by increasing its m^6^A modification levels and induces protective autophagy and mitophagy [43]. It seems that WTAP can play dual roles in the regulation of autophagy. Further research on the regulation of autophagy by WTAP is needed to decipher the exact role of it.

**Fig. 1:**
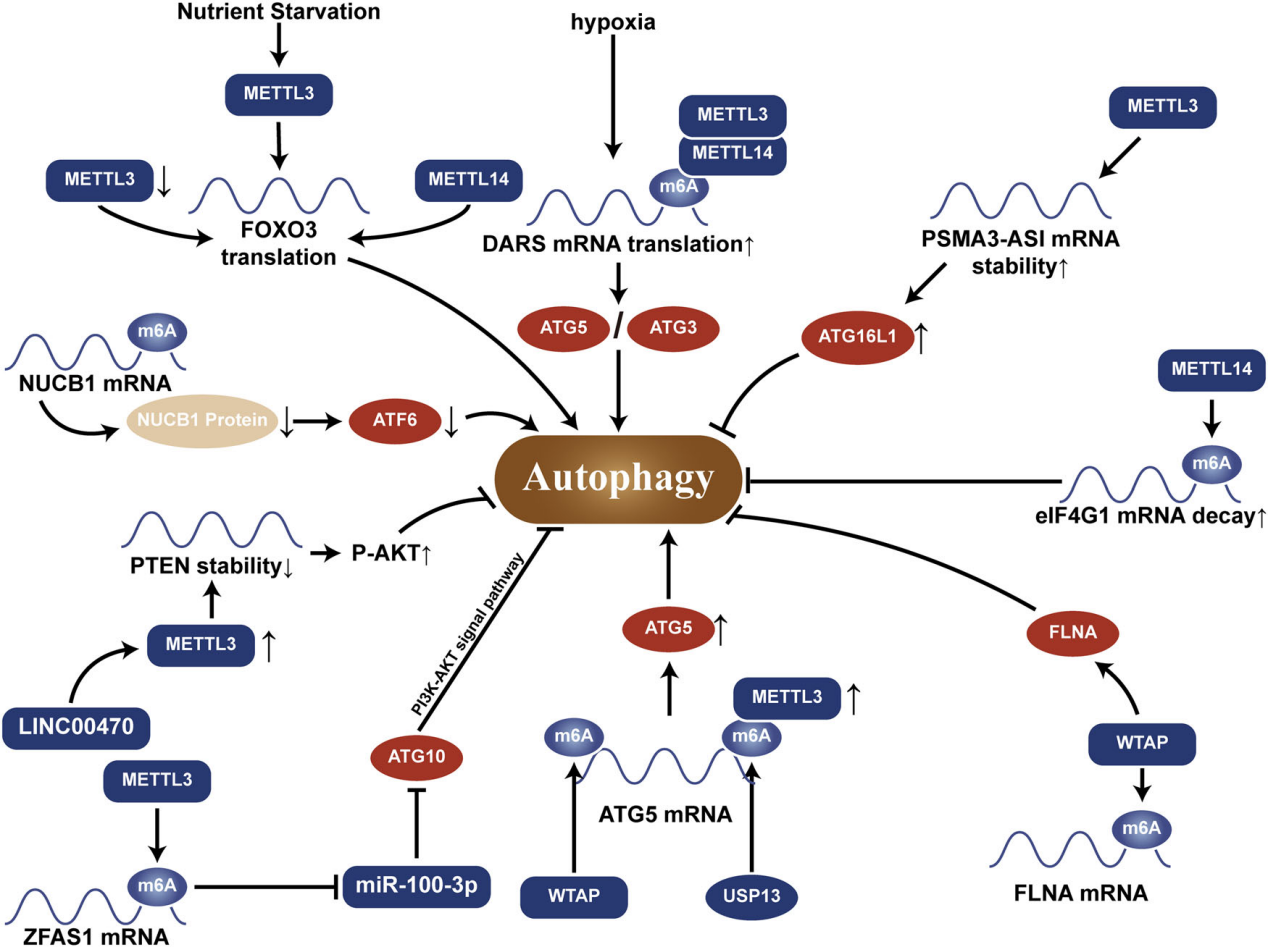
Mechanisms by which m^6^A writer regulates autophagy. Multiple writers affect autophagy by adding m^6^A modifications to target mRNAs and thereby altering their expression level.

Moreover, many studies have shown that the m^6^A methylation level of target gene mRNAs and their stability are not always positively correlated, and m^6^A modification may also negatively influence the stability or translational regulation of target gene mRNAs. Therefore, the regulatory relationship between m^6^A and autophagy is not entirely straightforward and may be related to the type of cell, the site of m^6^A modification, or the recognition of reader proteins (Fig. 1).

### M^6^A erasers and autophagy

Erasers are demethylases that correspond to writer proteins and are capable of removing the m^6^A modification from target genes through binding, indicating that m^6^A modification is a reversible and dynamic process [44, 45]. Fat mass and obesity-associated protein (FTO) was initially considered to be associated with human weight gain and obesity [46]. In 2017, for the first time, researchers revealed that FTO can play a crucial oncogenic role in acute myeloid leukemia (AML). It can promote cell proliferation and transformation and inhibit apoptosis in vitro, as well as significantly enhance the occurrence of leukemia in mice [47]. AlkB homolog 5(ALKBH5) belongs to the same AlkB family as FTO but with different substrates and functions and can either promote or inhibit the development of tumors [48]. In addition, studies suggest that ALKBH5 is involved in inducing cancer immune escape [49].

#### FTO

As a demethylase, FTO mainly plays a role in inhibiting autophagy. In contrast to the effect of writers, this effect involves removing m^6^A modifications from the mRNA of target genes, thereby affecting their expression and subsequently affecting autophagy [50, 51]. For instance, when FTO is bound by 5′-tRF-GlyGCC (a tRNA half-fragment and tRNA-derived small RNA fragment, TRF), its function is activated, leading to a reduction in the m^6^A modification level of the autophagic protein eIF4G1 mRNA, thus inhibiting autophagy [52]. However, FTO can also positively regulate autophagy. When FTO removes the m^6^A modification from the ULK1 mRNA, it becomes less susceptible to targeted degradation by YTHDF2, thereby promoting autophagy [53, 54].

#### ALKBH4/5

**Fig. 2:**
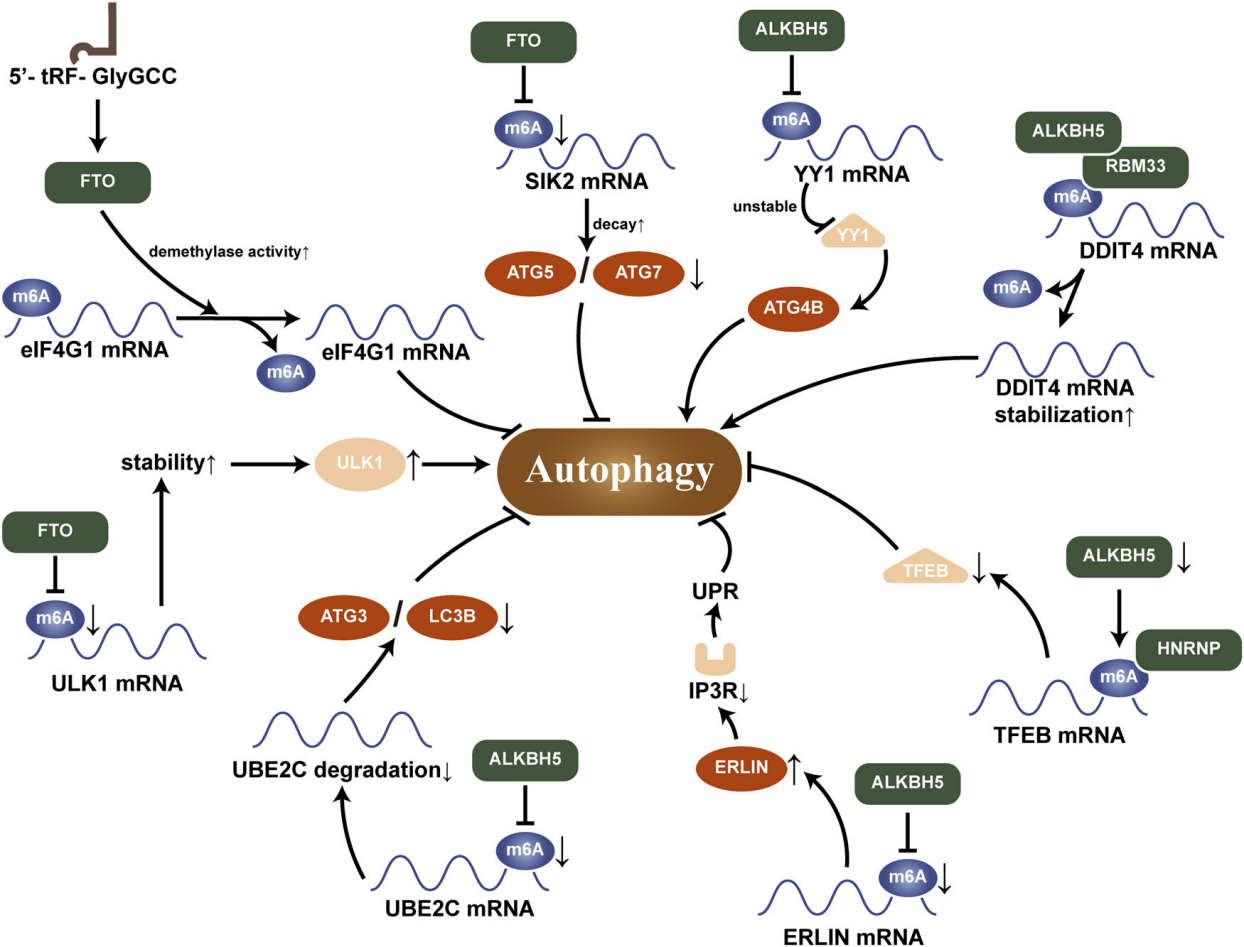
Mechanisms by which m^6^A eraser regulates autophagy. Altered levels of intracellular eraser can negatively affect the levels of target mRNA m^6^A modifications by altering their expression, which in turn affects autophagy.

ALKBH5 is a de novo demethylase. Similar in function to FTO, it regulates autophagy positively or negatively by affecting the stability and translation efficiency of mRNAs [55]. Yin-Yang 1 (YY1) is a ubiquitous transcription factor with multiple roles in tumorigenesis, functioning as both a transcriptional activator and repressor, and ALKBH5 can decrease the m^6^A levels of YY1 mRNA and suppresses the expression of ATG4B, which in turn inhibits autophagy [56, 57]. ALKBH5 can also demethylate ubiquitin-conjugating enzyme E2C (UBE2C) mRNA and decrease its stability, thereby directly and selectively inhibiting ATG3 and light chain 3 (LC3) and ultimately suppressing autophagy [55]. Additionally, ALKBH5 can inhibit the recognition by YTHDF3, thus inhibiting autophagy [58]. Conversely, ALKBH5 can remove m^6^A modifications and stabilize transcription factor EB (TFEB) or DNA damage-inducible transcript 4 (DDIT4) mRNA, thereby promoting autophagy [59, 60] (Fig. 2).

### m^6^A readers and autophagy

After m^6^A modification of mRNAs has been finished, specific reader proteins can be recruited and subsequently affect RNA processing, including translation and decay, thus regulating gene expression [24]. The YTH protein family are common m^6^A readers and includes YTHDF1/2/3 and YTHDC1/2. Studies have shown that YTHDF1 can promote the proliferation and metastasis of ovarian cancer cells by binding to the m^6^A-modified EIF3C gene and increasing the translation of eukaryotic translation initiation factor 3 subunit C (EIF3C) [61]. In contrast, YTHDF2 can recognize m^6^A modification on mRNA, promote RNA deadenylation, and ultimately mediate degradation of target mRNAs [62]. YTHDF3 can cooperate with YTHDF1 and YTHDF2 to promote the translation of the target mRNA [63]. YTHDC1 is the only protein of the YTH family that is located in the nucleus and mediates the selective splicing and nuclear export of premRNA [64]. YTHDC2 interacts with the small units of ribosomes that surround the mRNA, thereby directly participating in mRNA stability and translation [65]. Unlike the YTH family, as m^6^A readers, IGF2BPs can increase the stability of target mRNAs and promote their translation [9, 66].

#### YTH family

The YTH family includes YTHDF1/2/3 and YTHDC1/2 [67]. Upon binding, YTHDF1 increases the stability of BECN1 (Beclin 1, autophagy related) mRNA, thereby promoting autophagy [68]. YTHDF2 functions by promoting the degradation of NUCB1 mRNA [31]. Under nutrient-deficient conditions, YTHDF3 binds to the m^6^A hypermethylation set by METTL3 around the termination codon of FOXO3 mRNA and recruits eIFs to rapidly promote the translation of FOXO3 and thus increase autophagy [29, 69]. However, Zhang et al. reported that YTHDF2 can negatively regulate autophagy, which, as mentioned before, may be due to the characteristics of different target genes [54]. YTHDC1/2 are also members of the YTH family and play a role similar to that of YTHDF in promoting autophagy [42, 70].

#### IGF2BP

IGF2BPs, including IGF2BP1/2/3, are a class of important proteins regulating post-transcriptional stability and m^6^A modifications [71], among which IGF2BP2 is the most common. METTL3 and FTO deficiency can promote the stability of target gene mRNAs in an IGF2BP2-dependent manner, thereby promoting autophagy [50, 72]. Research has also shown that IGF2BP2 independently regulates the stability of its target gene metastasis-associated lung adenocarcinoma transcript 1 (MALAT1), consequently promoting the expression of its downstream target autophagy-related protein ATG12 [73]. IGF2BP3, on the other hand, regulates translation machinery associated 7 homolog (TMA7) via m^6^A modification, thus reducing autophagy [74].

#### Other readers

**Fig. 3:**
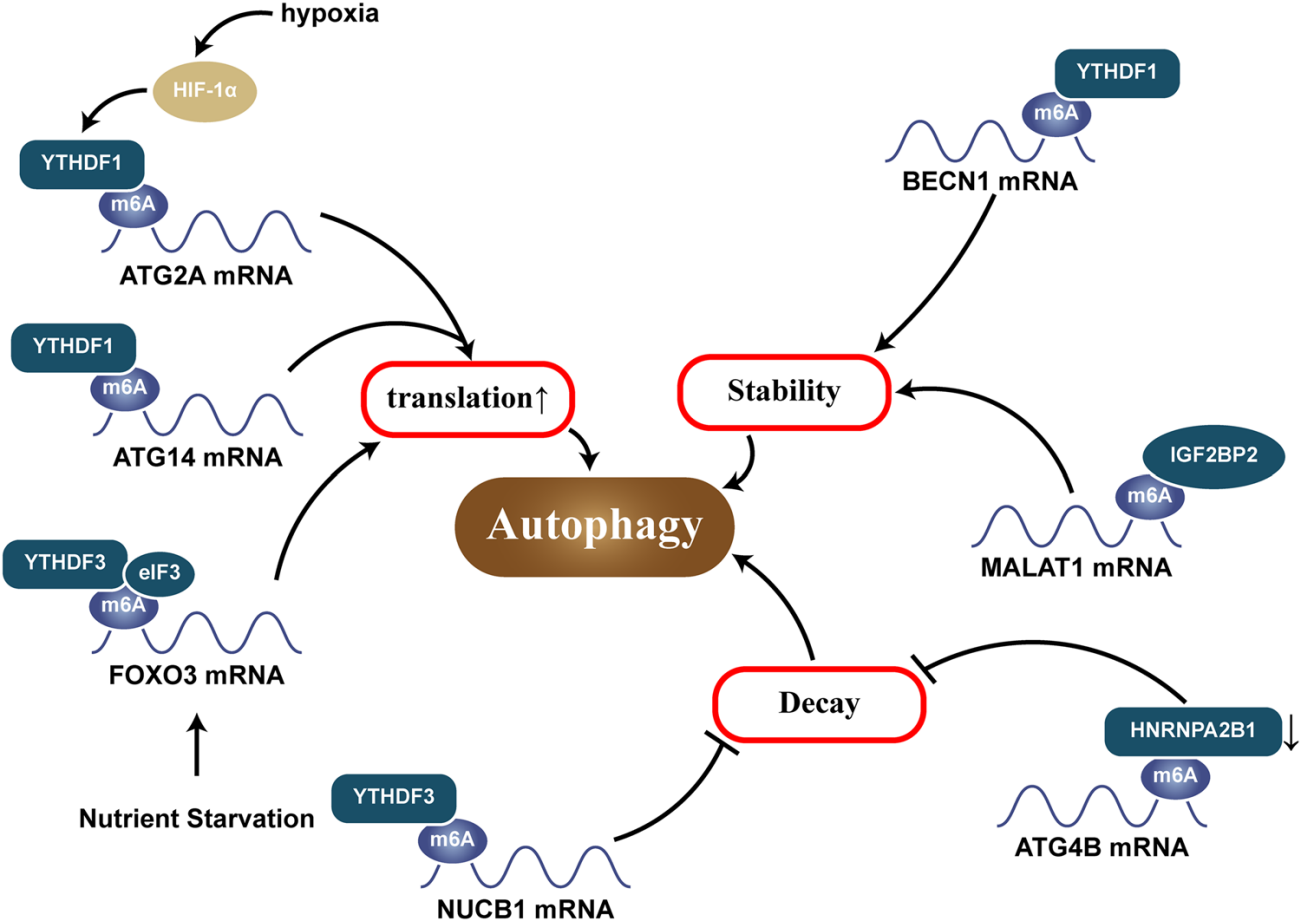
Mechanisms by which m^6^A reader regulates autophagy. Reader is affected by m^6^A modifications to bind to mRNAs, which in turn alters the stability of mRNA and their translation, which in turn affects autophagy.

In the heterogeneous nuclear ribonucleoproteins (HNRNP) family, HNRNPA2B1 acts as a reader, interacting with the m^6^A sites on ATG4B mRNA and accelerating its degradation, leading to a reduction in ATG4B expression and thereby inhibiting autophagy [75]. eIF3 is also an important reader [45]. Downregulates EIF3G can induce autophagy in colon cancer cells through inhibition of the mammalian target of rapamycin (mTOR) signaling pathway [76]. In contrast, studies have also shown that eIF3 can promote autophagy [77]. However, the specific underlying mechanisms have not yet been elucidated, and further research is needed (Fig. 3).

## Mechanism by which m^6^A modification regulates autophagy

Under physiological circumstances, autophagy is an evolutionarily conserved and self-degrading normal physiological process of cells that includes the induction and initiation of autophagy, the assembly and formation of autophagosomes, the fusion of autophagosomes with lysosomal membranes, and the degradation and recycling of autophagic contents within autolysosomes [78, 79]. Increasing evidence suggests that m^6^A modifications affect autophagy by participating in the induction of autophagy and influencing the formation, assembly and fusion of autophagosomes with lysosomes.

### m^6^A modification participates in autophagy induction and initiation

The PI3K/AKT/mTOR pathway is widely recognized as a regulator of autophagy, with mTOR being the central checkpoint for the negative regulation of autophagy [80]. In mammals, mTOR is divided into two different complexes according to their different functions, mTORC1 and mTORC2. Under nutrient-sufficient physiological conditions, the mTORC1 kinase applies an inhibitory phosphorylation tag on a key autophagy activation targets, ULK1, to prevent it from being activated by AMPK, which is a critical activator of autophagy [81]. In addition, inhibition of mTORC1 kinase allows ATG13 to anchor ULK1 to the pre-autophagosomal structure (PAS) and recruit almost all autophagy-associated proteins to initiate the autophagy program [78]. Studies have shown that m^6^A modifications increase the transcriptome stability of circMDK and target ATG16L1, thereby activating the PI3K/AKT/mTOR pathway in hepatocellular carcinoma cells [82]. The m^6^A modification of its downstream gene ULK1 can be removed by FTO, thus protecting it from targeted degradation by YTHDF2 and playing a role in cisplatin resistance in gastric cancer (GC) [53, 54].

mTORC1 also partially regulates autophagy by phosphorylating and inhibiting the nuclear translocation of the transcription factor TFEB, which drives the expression of lysosomal biogenesis-related genes and autophagy machinery genes [83]. The expression of TFEB mRNA is regulated by METTL3-mediated m^6^A modification in hypoxic cardiomyocytes [59]. Further research indicated that dephosphorylated TFEB can localize to the nucleus and activate autophagy and lysosomal biogenesis, thereby promoting tumor cell proliferation [84].

### m^6^A modifications affects autophagosome formation and assembly

The formation of mature autophagosomes is a complex and finely regulated process. In this process, the localization of the Atg12-Atg5-Atg16 complex to the PAS plays a key role in initiating and regulating the fusion of autophagic vesicle membranes [85], which is essential for the formation of autophagosomes. In particular, the presence of Atg5 and Atg16 (but not Atg12) in the complex is essential for its localization to the PAS, suggesting their irreplaceable roles in the maturation of autophagosomes [86, 87]. METTL3 can increase the m^6^A modification levels of USP13 mRNA and increase its stability of in gastrointestinal mesenchymal tumors. In OS, it is stabilized by USP13. Both of these modifications ultimately promote autophagy by stabilizing ATG5 expression via m^6^A modifications [35, 72]. In contrast, in chronic myelocytic leukemia (CML), LINC00470 has been shown to bind METTL3 and positively regulate the m^6^A modification levels of PTEN mRNA, consequently downregulating ATG5 expression and leading to chemotherapy resistance [32]. DARS-AS1/DARS can regulate the expression of ATG5 via m^6^A modification mediated by METTL3/14, indicating the development of autophagy in cervical cancer cells [34].

The elongation of the phagosome membrane also requires the generation of LC3-I/LC3-II, which is involved in the fusion of autophagosomes with lysosomes to form autolysosomes [88, 89]. As a marker protein of autophagic flux, LC3 has been used in a variety of experiments for the detection of autophagy. A study revealed that in multiple cancer cell lines, when ALKBH5 is silenced, the demethylation of m^6^A in ER lipid raft associated 1 (ERLIN1) mRNA is reduced, and an increase in the expression of the LC3B, a subtype of LC3, is observed, leading to increased autophagic flux [58].

In addition, research has also shown that m^6^A methylation modifications of AFAP1-AS1 promotes its translation into AFAP1-AS1 open reading frame 2 and inhibits the formation of autolysosomes by binding to the protein NIPSNAP1 on the inner mitochondrial membrane and trapping it, which is important for the malignant transformation of non-small cell lung cancer (NSCLC) cells [90].

### m^6^A modifications affects autophagy lysosome function

**Fig. 4:**
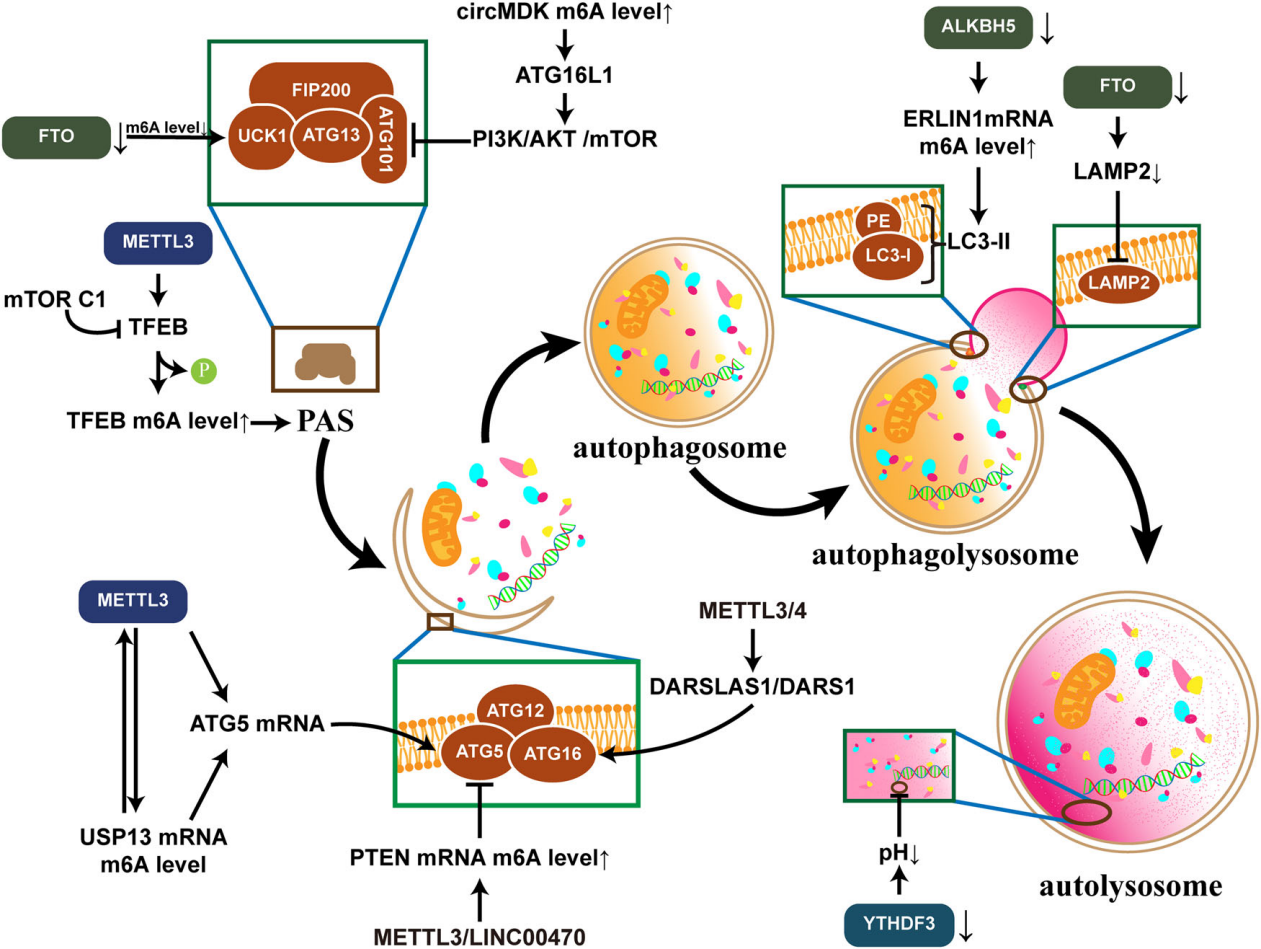
m^6^A modifications affect three main progresses, including the induction of autophagy, the assembly of autophagosomes, the binding of autophagosome membranes to lysosomal membranes, and degradation and recirculation of autophagosomal inclusions in autolysosomes, affecting the onset of autophagy.

After formation, autophagosomes rapidly migrate towards the centrosome, a lysosome hub, where they contact and fuse with the lysosomal membrane, mixing their contents to form autolysosomes [91, 92]. The hydrolytic enzymes in lysosomes help breakdown discarded cytoplasmic proteins and organelles within autophagosomes. Knockdown of the m^6^A-modified demethylation enzyme FTO significantly reduces the abundance of lysosome-associated membrane protein 2 (LAMP2), a key protein involved in mediating the fusion of lysosomes and autophagosomes, affecting their fusion [93]. The depletion of the m^6^A-binding protein YTHDF3 increases the pH of lysosomes, impairing cathepsin activity, hindering the formation of autophagosomes, causing lysosomal dysfunction, and affecting the function of autolysosomes, thereby damaging autophagic flux [29] (Fig. 4).

## m^6^A-mediated autophagy plays a dual role in tumor progression and therapy resistance

In 2018, a study by Jin et al. first demonstrated the mutual influence between m^6^A modification and autophagy and suggested that autophagy associated with m^6^A modification may play a role in tumorigenesis [53]. In subsequent studies, this hypothesis was confirmed, showing that m^6^A and autophagy have complex interactions that together regulate the malignant progression of tumors.

### Dual roles of m^6^A-mediated autophagy in malignant tumor progression

#### m^6^A-mediated autophagy promotes malignant tumor progression

Numerous studies have shown that autophagy-related proteins mediated by m^6^A are abnormally expressed in tumor tissues, leading to a series of changes that affect autophagy in tumors. For example, in head and neck squamous cell carcinoma (HNSCC), a novel m^6^A-binding protein, RBM33, can interact with ALKBH5 to regulate the stability of DDIT4 mRNA in a m^6^A-dependent manner, thereby promoting DDIT4-mediated autophagy, which maintains the tumorigenicity of HNSCC both in vitro and in vivo [60]. Similarly, colorectal cancer (CRC) cells rely heavily on glutamine to fulfill their metabolic needs during continuous proliferation, potentially leading to a glutamine-deficient environment. In contrast, inhibition of glutaminolysis can upregulate the demethylase FTO, thereby reducing the m^6^A modification of ATF4 mRNA, upregulating its expression and activating the transcription of DDIT4, deactivating mTOR signaling and inducing autophagy, which is beneficial for survival [94]. In conclusion, m^6^A-mediated autophagy may affect the occurrence and development of malignant tumors by regulating the oncogene DDIT4 [95].

Similarly, in OS, USP13 stabilizes METTL3 and its deubiquitination, further stabilizing ATG5 mRNA and activating oncogenic autophagy in OS [35]. In leukemia cells with nucleophosmin 1 (NPM1) mutations, FTO reduces m^6^A levels and enhances autophagy by upregulating the expression of TP53INP2 and promoting the LC3-ATG7 interaction, which is critical for the survival of NPM1-positive leukemia cells [51].

Given that m^6^A-mediated autophagy can promote pro-survival autophagy in tumors, could this further lead to processes that promote tumor progression, such as proliferation and invasion? As speculated, we found several studies demonstrating this phenomenon. In pancreatic cancer, ALKBH5 has been shown to directly modulate DDIT4-AS1, promoting the proliferation and metastasis of pancreatic cancer cells by inhibiting mTOR signaling [96]. Similarly, in GC, a ubiquitously expressed multifunctional zinc-finger transcription factor, Yin-Yang 1, is regulated by m^6^A modifications mediated by ALKBH5 and YTHDF1, activating the ATG4B-dependent autophagy pathway and thereby promoting the viability, proliferation, and migratory activity of GC cells [56]. In epithelial ovarian cancer (EOC), the methyltransferase WTAP stabilizes and positively regulates the expression of ULK1 by increasing its m^6^A modification levels in an IGF2BP3-dependent manner, which induces protective autophagy and mitophagy and promotes the proliferation and migration of tumor cells [43].

#### m^6^A-mediated autophagy inhibits malignant tumor progression

**Fig. 5:**
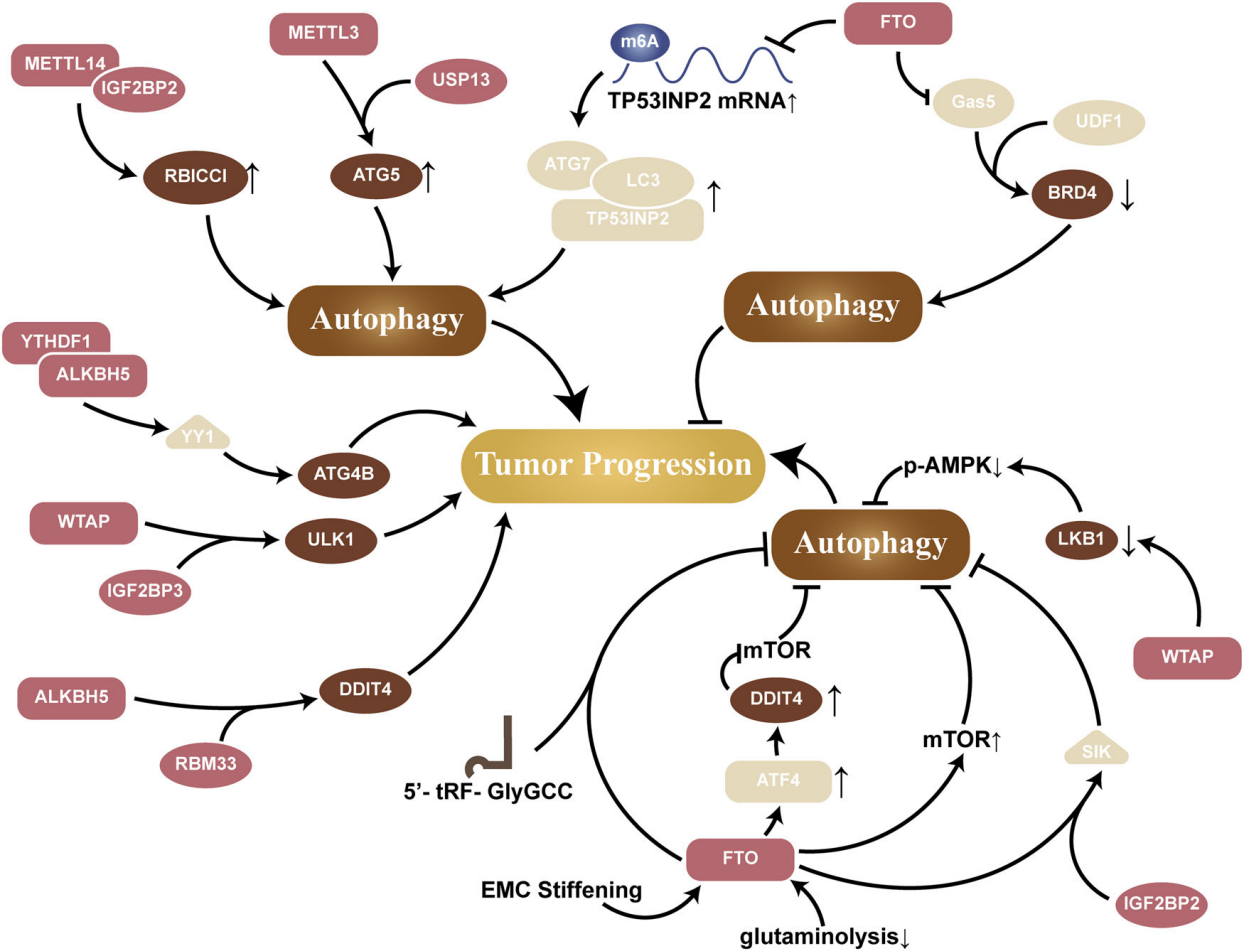
m^6^A-autophagy interactions jointly affect the occurrence and development of malignant tumors. On the one hand, m^6^A-autophagy interactions can alter autophagy genes or tumor-related genes, leading to the occurrence of pro-survival autophagy, and even further promoting tumor proliferation, invasion, and metastasis. On the other hand, in some tumors, an increase or decrease in autophagy flux can inhibit tumor proliferation, invasion, and metastasis, thereby inhibiting its malignant progression.

m^6^A-mediated autophagy can also play a role in suppressing tumor development and progression. Fu et al. demonstrated that the m^6^A demethylase FTO regulates Gas5 and inhibits the stability of BRD4 mRNA by interacting with up-frameshift protein 1 (UPF1), which promotes NSCLC autophagic cell death and thus suppresses NSCLC tumor growth [97]. In OSCC, the methyltransferase METTL14 targets and regulates RB1CC1 mRNA via a m^6^A consensus sequence in exon 15 in an IGF2BP2-dependent manner, increasing the autophagic flux of OSCC cells and inhibiting their proliferation, invasion, and migration. Moreover, an in vivo experiment showed that a lack of METTL14 promotes the growth of OSCC cells and their metastasis to cervical lymph nodes [38]. Similarly, Zhang et al. reported that the loss of the ECM affects m^6^A modifications, which may mediate alterations in mTOR protein abundance, modulating the mTORC1-autophagy pathway and thus contributing to EOC progression [98]. Given the important role that circulating tumor cells play in tumor metastasis [99], the relationship between autophagy due to ECM shedding and EOC metastasis also deserves further exploration. J. Guo et al. reported that inhibition of m^6^A-regulated autophagy phenotypically prevented NSCLC progression [55]. In addition, the inhibition of autophagy can also inhibit the progression of malignant tumors. For example, inhibition of WTAP reduces the m^6^A modification level of LKB1 mRNA and increases LKB1 Mrna expression, thereby increasing the phosphorylation of AMPK, which increases autophagy and inhibits hepatocellular carcinoma (HCC) cell proliferation to some extent [41]. Similarly, in breast cancer (BC), the inhibition of 5′-tRF-GlyGCC binding to FTO can promote autophagy, which increases the m^6^A level of eIF4G1 in BC cells and inhibits growth and metastasis [52]. This may be because autophagy can contribute to the maintenance of cellular genetic stability by removing damaged DNA and defective organelles [100], and the promotion of such autophagy can inhibit tumor progression (Fig. 5) (Table 1) [12, 18].

**Table 1:** Mechanisms by which the M^6^A-autophagy axis regulates tumor progression.

m6A regulator		Autophagy effector	Regulation of the main autophagy effector	Association between m6A and autophagy	Tumor	Autophagy function on tumor progression	Reference
Writer	METTL3	ATG5	Increase m6A level	Positive	OS	Promote	[35]
	METTL3 &METTL14	DARS/ATG5/ATG3	Increase m6A level	Positive	CESC	Promote	[34]
	METTL14	RB1CC1	Increase m6A level	Positive	OSCC	Inhibit	[37]
		eIF4G1	Increase m6A level	Positive	OSCC	Inhibit	[36]
	WTAP	FLNA	Increase m6A level	Negative	COAD	Inhibit	[39]
		LKB1/AMPK	Increase m6A level	Negative	HCC	Promote	[40]
		ULK1	Increase m6A level	Positive	EOC	Promote	[94]
		ATG5	Increase m6A level	Positive	HCC	Inhibit	[41]
Eraser	FTO	eIF4G1	Decrease m6A level	Negative	BC	Promote	[50]
		TP53INP2/ LC3/ATG7	Decrease m6A level	Positive	AML	Promote	[49]
		Gas5	Decrease m6A level	Positive	NSCLC	Inhibit	[95]
		SIK2	Decrease m6A level	Negative	ccRCC	Promote	[48]
		ATF4/DDIT4/mTOR	Decrease m6A level	Positive	CRC	Promote	[91]
		mTORC1	Decrease m6A level	Negative	EOC	Inhibit	[96]
	ALKBH5	UBE2C/ATG3/LC3	Decrease m6A level	Negative	NSCLC	Inhibit	[53]
		YY1/ATG4B	Decrease m6A level	Negative	GC	Inhibit	[54]
Reader	YTHDF1	ATG2A/ATG14	Increase mRNA translation	Positive	HCC	promote	[111]
		YY1/ATG4B	Increase mRNA stability	Negative	GC	inhibit	[54]
	YTHDC2	ATG5	Increase mRNA translation	Positive	HCC	Inhibit	[41]
	IGF2BP2	MALAT1/ATG12	Increase mRNA stability	—	NSCLC	Promote	[41]
		SIK2	Increase mRNA stability and translation	Positive	ccRCC	Inhibit	[48]
	IGF2BP3	TAM7	Increase mRNA stability	Negative	LSCC	Promote	[71]
	RBM33& ALKBH5	DDIT4	Decrease m6A level and increase mRNA stability	Positive	HNSCC	Promote	[57]
	HNRNPA2B1	ATG4B	Facilitate decay	Positive	BC	Promote	[72]

### Dual roles of m^6^A-mediated autophagy in tumor therapy resistance

#### m^6^A-mediated autophagy promotes the development of therapeutic resistance in tumors

Several studies have demonstrated that m^6^A-mediated autophagy is one of the important pathways of chemotherapy drug resistance in malignant tumors and regulates the chemosensitivity of a variety of malignant tumors by interacting with upstream or downstream genes.

Cisplatin is one of earliest and most effective metal-based chemotherapy drugs and is widely regarded and used as a potent agent for treating various solid tumors [101]. The resistance of tumors to cisplatin depends on various factors, including m^6^A modifications and autophagy. In lung squamous cell carcinoma (LSCC), IGF2BP3-mediated m^6^A modification of TMA7 regulates autophagy via the UBA2-PI3K pathway, promoting resistance to cisplatin in LSCC cells, whereas the autophagy inhibitor rapamycin sensitizes cells to cisplatin treatment [74]. In seminoma, overexpression of METTL3 upregulates ATG5 expression and m^6^A modification levels, inducing autophagy and spermatogonial drug resistance [102]. In GC, upregulated expression of FTO promotes cisplatin resistance in GC cells by regulating YTHDF2-associated ULK1 expression, facilitating autophagy-induced cisplatin resistance [54]. Therefore, inhibiting autophagy could be a strategy for alleviating cisplatin resistance, thereby improving therapeutic outcomes and clinical results.

Moreover, in GC, ARHGAP5-AS1 promotes the upregulation of its target gene ARHGAP5 and regulates the m^6^A-autophagy axis, thereby promoting drug resistance in tumor cells [103]. For gastrointestinal mesenchymal stromal tumors (GIST), Gao et al. first discovered that USP13, which is regulated by METTL3-IGF2BP2, stabilizes ATG5 in a PAK1-dependent manner, increasing its expression and potentially inducing protective autophagy that enhances GIST tolerance to imatinib. Thus, inhibiting ATG5 could be considered a strategy to suppress resistance [72]. In sorafenib-resistant HCC cells, METTL3 is significantly downregulated, leading to FOXO3 degradation, thus promoting autophagy-induced resistance to sorafenib in HCC [30]. Regulation of DCP2 by METTL3 in small cell lung cancer (SCLC) alters the level of chemotherapeutic drug-induced mitochondrial damage, subsequently leading to chemoresistance. The use of the METTL3 inhibitor STM2457 to reverse the chemotherapy resistance of SCLC cells, both in vitro and in vivo, is expected to become a new adjuvant therapy drug [104].

#### m^6^A-mediated increases in tumor sensitivity to drugs

**Fig. 6:**
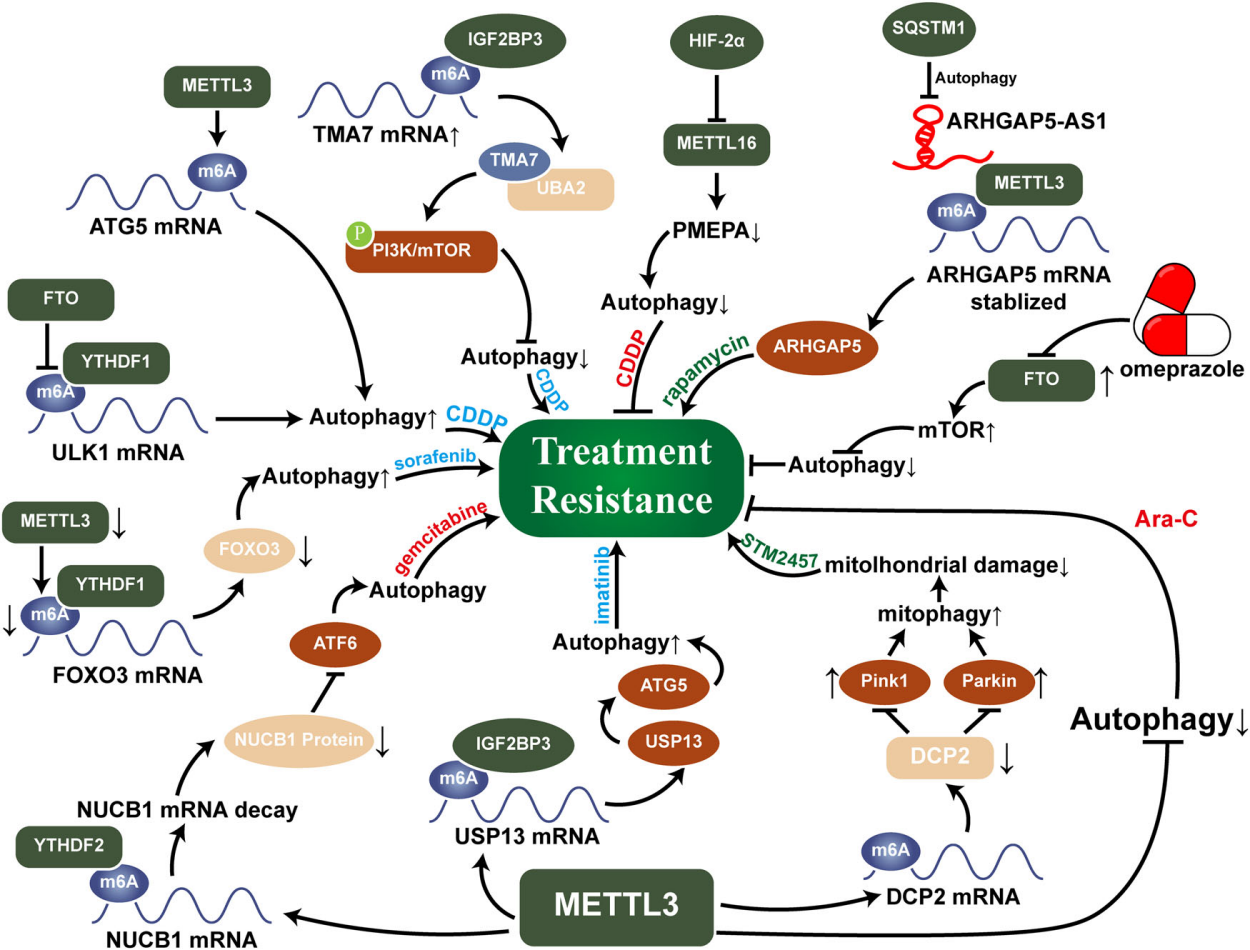
Mechanism diagram of m^6^A-autophagy-mediated tumor treatment resistance. Changes in the levels of m^6^A regulators, the methylation levels of resistance-related genes, and the expression of autophagy-related genes in tumor tissues may all lead to increased or decreased tumor responsiveness to therapeutic drugs. Therefore, it may be possible to reverse tumor drug resistance by targeting these targets. In the figure, drugs marked in red indicate that the tumor has increased sensitivity to them, drugs marked in blue indicate that the tumor has decreased sensitivity to them, and drugs marked in green can act on this pathway to reverse the tumor’s drug resistance.

m^6^A-mediated autophagy has a dual effect and can also increase tumor sensitivity to drugs. METTL16, through the HIF-2α-PMEPA1-autophagy axis, significantly inhibits the proliferation of bladder cancer (BLCA) cells in vitro and in vivo via m^6^A modification, increasing the sensitivity of these cells to cisplatin. However, METTL16 is significantly downregulated in human BLCA tissues; thus, increasing METTL16 is a potential therapeutic target for overcoming resistance in BLCA [105]. Omeprazole-induced FTO inhibition enhances the activation of the mTORC1 signaling pathway and inhibits the pro-survival effect of autophagy, thereby increasing the antitumor efficacy of chemotherapeutic drugs in GC cells and leading to a synergistic tumor-suppressive effect [106]. METTL3 depends on YTHDF2 to promote the m^6^A modification of NUCB1, thereby regulating ATF6, blocking gemcitabine (GEM)-induced autophagy and enhancing the anti-tumor effect of GEM [31]. Similar findings have also been observed in nonsolid tumors as well. In AML, METTL3 promotes the drug sensitivity of tumor cells to Ara-C by inhibiting autophagy in AML cells [107] (Fig. 6) (Table 2).

**Table 2:** Role of the m^6^A-mediated autophagy axis in tumor therapy resistance.

Upstream modulator	m6A regulator			Effector	m6A level of autophagy effector	Association between m6A and autophagy	Tumor	Function on tumor therapy resistance	Reference
	Writer	Eraser	Reader						
/	METTL3	/	YTHDF1	FOXO3	Decrease	Positive	HCC	Sorafenib resistance	[30]
/		/	YTHDF2	NUCB1	Decrease	Negative	PDAC	Gemcitabine enhancement	[31]
/		/	IGF2BP2	USP13	Increase	Positive	GIST	Imatinib resistance	[69]
β-elemene		/	/	ATG5、ATG7、LC3B、SQSTM1 etc.	Decrease	Positive	NSCLC	Reverse gefitinib resistance	[116]
/		/	/	DCP2	Increase	Positive	SCLC	Chemoresistance	[103]
LINC00470		/	/	PTEN	Increase	Negative	CML	Chemoresistance	[32]
/		/	/	ATG7、FOXO1、FOXO3、MAP1LC3A、BCL2 etc.	/	Negative	AML	Enhancing Ara-C sensitivity	[104]
/		/	/	ATG5	Increase	Positive	TGCT	Cisplatin resistance	[100]
/		/	/	ARHGAP5	Increase	Positive	GC	Chemoresistance	[101]
/	METTL16	/	/	PMEPA1	Increase	Positive	BLCA	Enhancing cisplatin sensitivity	[111]
/	/	FTO	YTHDF2	ULK1	Decrease	Positive	GC	Cisplatin resistance	[52]
Omeprazole	/	FTO	/	DDIT3	Increase	Positive	GC	Enhancing anti-tumor drugs sensitivity	[115]
/	/	/	HNRNPA2B1	ATG4B	/	Positive	BC	Reverse olaparib resistance	[72]
/	/	/	IGF2BP3	TMA7	/	Negative	LSCC	Cisplatin resistance	[71]

### The m^6^A-autophagy axis is involved in the signaling pathway network of tumors

A number of studies have demonstrated that m^6^A does not solely act on autophagy but that autophagy can also influence m^6^A modification by affecting its regulators, which play a role in the development and progression of malignant tumors. HPV-E6 inhibits the degradation of the RBM15 protein in cervical cancer cells via autophagy, subsequently affecting the m^6^A modification of c-myc mRNA [108]. Recently, LINRIS was shown to inhibit the ubiquitination of IGF2BP2, thereby preventing its degradation via the autophagy-lysosomal pathway [109]. Ultraviolet B (UVB) radiation plays an important role in the development of skin cancer. Autophagy, through downregulation of the methyltransferase METTL14 via NBR1, reduces m^6^A modification levels, thus decreasing UVB damage repair and inducing tumorigenesis in the skin [110]. m^6^A modification of RNA reduces the proliferation and viability of melanoma cells. Thus, metabolic stress induces FTO via autophagy and the NF-κB pathway. In addition, FTO removes m^6^A tumor-suppressive m^6^A modifications in melanoma, thus playing a tumorigenic role [111].

**Fig. 7:**
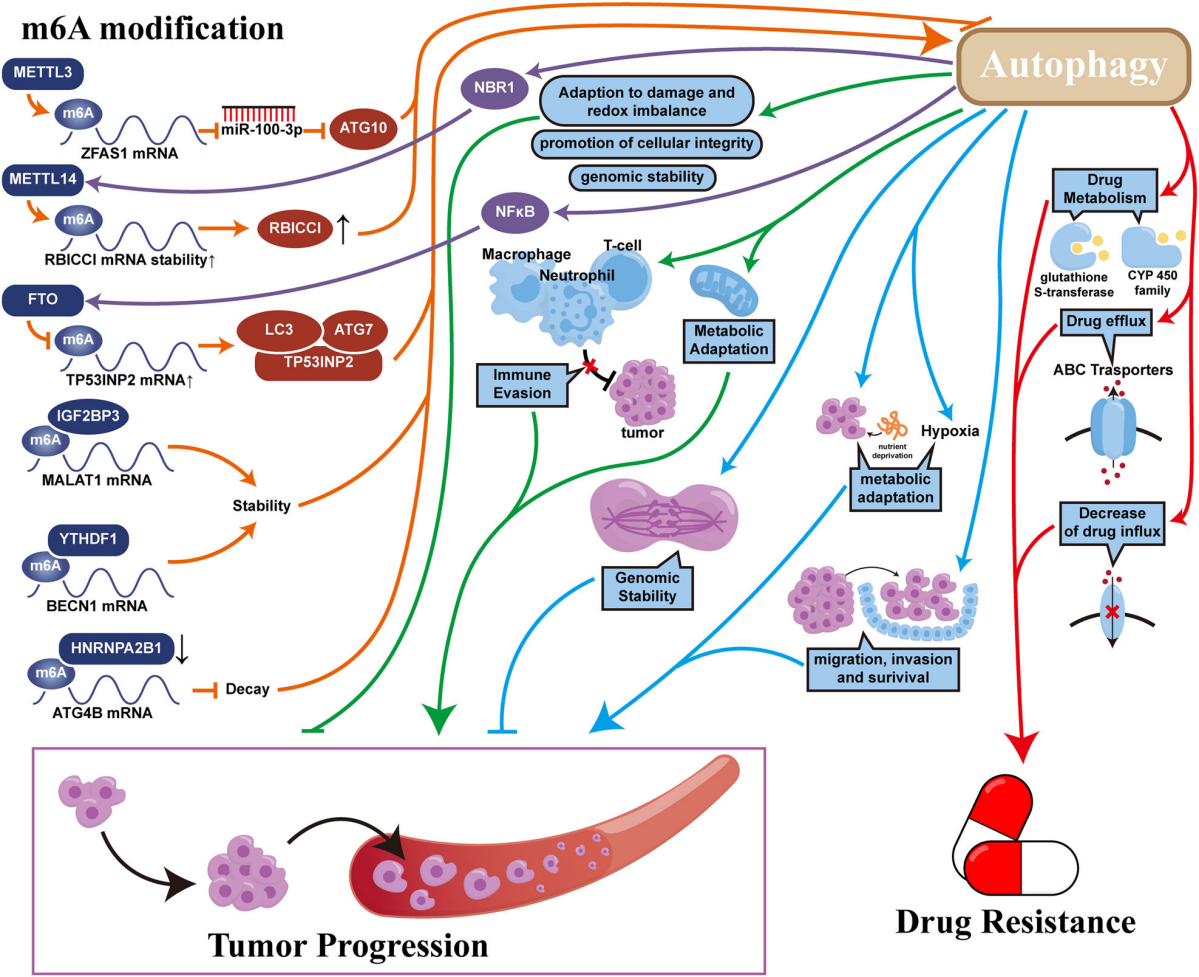
Overview of m^6^A autophagy axis involved in tumor signaling pathway network. m^6^A modification and autophagy form an interactive network, altering multiple physiological processes of tumor cells and ultimately affecting the malignant progression and treatment resistance of tumors.

Therefore, we found that m^6^A modifications are important for the regulation of autophagy and that autophagy can regulate m^6^A modifications by affecting the activity of regulators and maintaining normal levels of methyltransferases, demethylases, and m^6^A selective binding proteins, thus preserving normal physiological states. Thus, we found that m^6^A and cellular autophagy interact with each other to form a regulatory network. The findings in keratinocytes by Y. H. Cui et al. corroborate our conclusion. UVB damage to such cells may induce skin tumors. During the UVB damage repair process, low levels of arsenic upregulated FTO, reduce m^6^A levels in NEDD4L, decrease mRNA stability, and subsequently decrease mRNA and protein levels, inducing malignant transformation and tumorigenesis. Moreover, arsenic stabilizes the FTO protein by inhibiting p62-mediated selective autophagy. Upregulated FTO, in turn, can inhibit autophagy, thus forming a positive feedback loop to maintain FTO accumulation and amplify its role in inducing malignant tumors [112] (Fig. 7).

## Value of the m^6^A-autophagy axis in tumor diagnosis and therapy

### m^6^A-autophagy axis-related proteins are potential molecular markers for tumor diagnosis

The aberrant expression of m^6^A-modified regulators, including writers, erasers, and readers is found in numerous tumor types and is closely associated with the diagnosis and prognosis of tumors. Using the METTL family as an example, the analysis of data obtained from The Cancer Genome Atlas (TCGA) database revealed that increased expression of METTL16 is significantly correlated with tumor size, lymph node metastasis, distant metastasis, and clinical stage grading in CRC. Further studies in clinical samples have shown that the expression of METTL16 is significantly elevated in CRC tissues and is correlated with a poor prognosis, suggesting that METTL16 may be a promising diagnostic biomarker for CRC [113]. In addition, the high expression of METTL3 in small cell lung cancer (SCLC) is associated with poor overall survival [104], while the reduced expression of METTL14 in OSCC correlates with advanced T stage, poor differentiation, and lymph node metastasis [38], suggesting that METTL14 is a biomarker of poor prognosis. In contrast, high expression of the reader YTHDF1 and its related proteins ATG2A and ATG14 in HCC is associated with poor prognosis, demonstrating their potential as prognostic indicators and bases for the development of therapeutic strategies [114]. Some genes and their products involved in the m^6^A-autophagy axis are also associated with the prognosis of malignant tumors. For example, ARHGAP5-AS1/ARHGAP5 plays a role in activating the m^6^A-autophagy axis in GC, and its upregulation is associated with poor prognosis in patients with GC, making it a potential prognostic marker [103].

More interestingly, some researchers have also developed prognostic prediction models based on these related genes. Yu et al. developed a prognostic prediction model for lung squamous cell carcinoma based on seven m^6^A-associated autophagy genes using data from the TCGA database, which can distinguish between high-risk and low-risk patient groups [115]. Similarly, Chen et al. developed a prognostic scoring model for esophageal squamous cell carcinoma using six m^6^A-associated autophagy genes [116]. However, as both models were derived from online databases, their validity in the real world remains to be determined by further studies.

### m^6^A-autophagy axis-related proteins are expected to be important molecular targets for tumor therapy

m^6^A-regulated autophagy can also have an effect on Programmed Cell Death Protein 1 (PD-1)/ Programmed Cell Death Ligand 1 (PD-L1) therapy. In melanoma, metabolic stress upregulates FTO via autophagy and the NF-κB pathway. The expression of PD-1 is positively regulated by FTO-YTHDF2, and therefore, inhibition of FTO can enable mice to develop antimelanoma responses to PD-1 immunotherapy; perhaps using FTO inhibitors during treatment could increase the effectiveness of immunotherapy [111]. For chemotherapeutic drugs, in CRC, LINC01615 is upregulated by autophagy-regulated METTL3 and inhibits oxaliplatin efficacy by recruiting hnRNPA1. Therefore, targeting the autophagy-METTL3-LINC01615 axis could reduce oxaliplatin resistance [117].

Given that the m^6^A-autophagy axis plays a critical role in therapeutic resistance in tumors, modulation of this pathway would be beneficial for inhibiting tumor therapeutic resistance in tumors. Research has shown that the proton pump inhibitor omeprazole can downregulate FTO and inhibit pro-survival autophagy in GC, thereby increasing the efficacy of chemotherapeutic agents and synergizing with antitumor medications [106]. Consequently, the combined use with omeprazole may be a promising strategy to overcome chemotherapy resistance in patients with GC. In lung cancer, for NSCLC, METTL3 promotes autophagy by upregulating the expression of LC3B, ATG5, and ATG7, protecting drug-resistant cells from death and leading to the development of resistance to gefitinib. This effect can be reversed by β-elemene, which reduces lysosomal acidification, potentially serving as a promising anti-resistance adjunctive therapy [118]. In SCLC, the regulation of DCP2 by METTL3 alters the level of chemotherapeutic drug-induced mitochondrial damage, leading to chemotherapy resistance. However, a new, highly selective, oral METTL3 inhibitor, STM2457, abrogates the resistance of SCLC cells to chemotherapy both in vitro and in vivo, indicating its potential for treating chemotherapy-resistant SCLC patients [104]. In BC, downregulation of the reader HNRNPA2B1 can reduce BC cell proliferation, increase autophagic flux, and partially abrogate resistance to olaparib, potentially becoming an important target for increasing chemotherapy sensitivity [75].

## Conclusion and prospects

In this review, we summarize the regulatory roles of m^6^A modification regulators, including writers, erasers and readers, in the m^6^A modification and expression of cellular autophagy-related genes; outline the roles of m^6^A modification in the initiation of autophagy, the assembly of autophagosomes, and the functioning of lysosomes in tumor cells; and summarize the functions and mechanisms of the m^6^A-autophagy axis as well as autophagy-m^6^A feedback regulation in malignant progression and therapeutic resistance in tumors, thus forming a network of m^6^A-autophagy-tumor interactions. We also noted that autophagy plays dual roles in promoting or suppressing tumor progression and therapeutic resistance, a function that differs from that of other forms of programmed cell death in tumors. The specific effects are dependent on the type of tumor cells, stress state, m^6^A modification site, and types of readers. These findings deepen our understanding of the complex regulatory relationships among m^6^A modification, autophagy, and cancer. Additionally, based on the expression characteristics and important functions of molecules related to the m^6^A-autophagy axis in the malignant progression and therapeutic resistance of tumors, we summarized the clinical value and application prospects of m^6^A-autophagy axis-related molecules in the clinical diagnosis and treatment of cancer. Therefore, targeting the m^6^A-autophagy axis will undoubtedly become an important molecular target and a potential molecular strategy for cancer diagnosis and treatment in the future.

Moreover, we must recognize that the regulatory relationships among m^6^A modifications, autophagy and cancer are highly complex, and it is still a great challenge to fully identify their intricate networks and applications in cancer diagnosis and treatment. First, research on the regulation of m^6^A modification factors by the cellular autophagy process is sparse and not very thorough, and information on feedback regulation is limited to very few m^6^A regulatory factors. Second, cellular autophagy has dual roles in tumor cell survival and malignant progression. Determining under what conditions it promotes or inhibits cancer and the mechanisms of its fine regulation still need further clarification. Hence, it is currently difficult to draw a comprehensive conclusion on the complex regulatory relationship between the two. Finally, activators or inhibitors targeting m^6^A regulators have not yet been applied in clinical tumor treatment, and their safety and efficacy still need further evaluation. Additionally, the low specificity of m^6^A writers, erasers, and readers is another major impediment to the application of m^6^A regulators. Therefore, more in-depth studies are needed to confirm the regulatory relationships among m^6^A modification, autophagy, and cancer in the future.
